# Identification of Novel Quantitative Trait Loci and Candidate Genes Associated with Grain Yield and Related Traits Under Low-Light Stress Conditions in Rice

**DOI:** 10.3390/biom15101388

**Published:** 2025-09-29

**Authors:** Soumya Mohanty, Swagatika Das, Darshan Panda, Nalini Kanta Choudhury, Baneeta Mishra, Ranjan Kumar Jena, Rameswar Prasad Sah, Anil Kumar Chandrappa, Devanna Basavantraya Navadagi, Reshmi Raj K.R., Awadhesh Kumar, Sharat Kumar Pradhan, Sanghamitra Samantaray, Mirza Jaynul Baig, Lambodar Behera

**Affiliations:** 1 ICAR-Central Rice Research Institute, Cuttack 753006, Odisha, India; smmohanty.mohanty9@gmail.com (S.M.);; 2 Indian Council of Agricultural Research, Pusa, New Delhi 110012, India

**Keywords:** abiotic stress, candidate genes, grain yield, hub gene, low light, quantitative trait loci, rice

## Abstract

Low light intensity is a major abiotic stress that severely affects rice yields, particularly in India and Southeast Asia, causing yield reductions of 35–40% during the wet season compared to the dry season. Tolerant rice genotypes exhibit adaptive changes at anatomical, physiological, biochemical, and molecular levels under low-light stress, enabling higher yields compared to susceptible varieties. Our study identified 20 novel QTLs associated with grain yields and nine related traits under low-light and control (normal)-light conditions, using a recombinant inbred line (RIL) population derived from the cross between the low-light-tolerant variety Swarnaprabha and the low-light-susceptible variety IR8. Across the *Kharif* seasons of 2019 and 2021, 33 stable QTLs were identified, with 11, 13, and 9 QTLs specific to low-light, normal-light, and both conditions, respectively. Of these, Swarnaprabha contributed 28 QTLs, while five were contributed by IR8. Notably, the study identified 11 and 9 novel QTLs under low-light and both conditions, respectively. Three hotspot regions on chromosomes 1, 4, and 8 were identified. These regions harbored 10 novel QTLs and revealed twenty candidate genes, out of which three key hub genes, *OsAUX1*, *OsSBDCP1*, and *OsNPF5.16*, were identified. These hub genes are involved in hormone signaling, starch metabolism, and nitrogen metabolism, respectively. A comprehensive expression analysis of these genes indicated that they are linked to low-light tolerance, offering deeper insights into the genetic and molecular mechanisms underlying low-light resilience. These findings provide valuable genomic resources and potential markers for breeding programs for improving rice productivity under low-light conditions.

## 1. Introduction

Light is a fundamental factor influencing plant growth, development, and productivity, serving as the primary energy source for photosynthesis [1]. An adequate light intensity is crucial for optimizing crop yields and ensuring food security. Low-light (LL) stress, characterized by insufficient light intensity, significantly impacts rice (*Oryza sativa* L.), a staple food for over half of the global population [2,3]. LL stress reduces the tiller number, grain number, spikelet fertility, panicle number, grain weight, and dry matter production, causing substantial yield losses [4,5]. During critical growth stages such as tiller development, panicle differentiation, and grain filling, LL conditions can reduce grain yields by 35–40% by impairing the photosynthetic efficiency [6,7]. Addressing LL stress is essential for maintaining rice yields in affected regions. Environmental factors such as cloudy skies, prolonged cloudiness, and heavy rainfall during the rainy season contribute to LL conditions [8]. The eastern and north-eastern parts of India, particularly the north-eastern hill region (NEHR), are especially vulnerable due to the unpredictable nature of monsoons and persistent cloud cover [9]. Southeast Asia and China also face LL stress due to similar weather patterns, exacerbated by air pollution and densely planted rice fields [10]. While rice yields its highest potential during the *Rabi* season with abundant sunlight, the reliance on rainwater for irrigation leads to rice cultivation predominantly during the *Kharif* (rainy) season [11], despite the yield reductions caused by LL conditions. LL significantly impacts the crop morphology, grain yield, and grain quality. Under LL, rice plants often show increased plant heights and leaf areas but reduced spikelet numbers and filled grains [2,12]. In LL-tolerant genotypes, the panicle length often increases, although LL during the reproductive period reduces the panicle dry weight [13,14]. Additionally, it also decreases the number of panicles before heading and reduces the number of filled grains and the 1000 grain weight after heading [3,15]. Reduced photosynthesis under LL conditions diminishes the transport of assimilates to developing grains, impacting the seed set and overall grain production [16,17]. LL negatively affects the grain quality, leading to undesirable appearances and reduced milling characteristics [18,19]. Physiological and biochemical changes induced by LL stress ultimately cause decreased crop productivity. Optimal photosynthesis and grain filling in rice are achieved under light intensities ranging from 700 to 1000 μmol m^−2^s^−1^ of photosynthetically active radiation (PAR) [20,21]. A drop in light intensity below 700 μmol m^−2^s^−1^ reduces the photosynthetic efficiency, contributing to yield reductions under LL [22,23]. The activity of photosystem II (PSII) decreases with declining light intensity, reducing the electron transport (ETR) and Calvin cycle efficiency [24]. This diminishes the generation of photosynthates, which are critical for growth, impacting the seed set and total grain production [25]. LL stress also affects the synthesis of non-structural carbohydrates such as sucrose, lignin, and cellulose, which are essential for maintaining stem strength and preventing lodging [26]. Recent molecular studies have highlighted the transcriptome, microRNA (miRNA), and small RNA (sRNA) dynamics in plants under LL conditions, revealing complex regulatory networks that enable plants to acclimatize to shaded environments. Transcriptomic analyses have shown significant changes in gene expression patterns, including the downregulation of genes associated with light harvesting and the upregulation of genes involved in UV light protection when transitioning from LL to high light [27]. Prolonged LL exposure leads to the degradation of hyponastic leaves 1 (*Hyl1*), allowing plants to adapt to shade, while light restoration activates *Hyl1*, promoting microRNA production and maximizing light absorption [28]. Additionally, genes associated with chlorophyll a/b-binding proteins (CAB), light-harvesting complexes (LRP), and photosystem I and II complexes were upregulated in the LL-tolerant rice genotype Swarnaprabha after prolonged shade exposure, compared to the LL-susceptible genotype IR8 [29]. Several miRNAs, such as miR156, miR160, miR397, and miR399, are implicated in rice responses to LL stress [30]. Sekhar et al. [31] identified both novel and known miRNAs in rice, including osa-novmiR1, osa-novmiR2, osa-miR166c-3p, and osa-miR530-3p, which regulate genes involved in photosynthesis and metabolic pathways, influencing rice’s adaptation to LL conditions. Identifying quantitative trait loci (QTLs) associated with LL tolerance is crucial in crop genetics, particularly for rice. Despite extensive studies, specific QTLs and genes conferring shade tolerance remain largely unidentified. Recent research has begun to address this gap. For instance, Wang et al. [32] identified YGL9, a novel chloroplast transit peptide protein implicated in chloroplast development, marking an advancement in understanding LL tolerance. Dutta et al. [33] identified six markers associated with improved rice performance under LL, offering insights for marker-assisted breeding strategies. Ganguly et al. [34] and Saha et al. [35] have highlighted the genetic and molecular mechanisms enabling rice to adapt to LL conditions. Ganguly et al. [34] identified the significant QTLs *qPNLL1.1* and *qGYLL1.1* that contribute to yield enhancements under LL stress by leveraging genetic variability within rice. They identified several QTL regions that are consistently involved in light-responsive pathways, enhancing our understanding of the genetic basis of LL tolerance. Saha et al. [35] indicated the role of the A400/A1800 ratio as a rapid phenotyping tool for LL tolerance. Their findings demonstrate that this ratio is strongly correlated with the photosynthetic efficiency and photoprotection capabilities of rice, offering a practical approach for breeders to screen for LL tolerance effectively. Recent advancements have enhanced our understanding of the genetic mechanisms governing LL tolerance in crops, offering valuable insights for developing tolerant varieties. Khumaida et al. (2015) observed higher expression levels of the *CAO3–4* gene in shade-tolerant soybean genotypes compared to shade-sensitive counterparts, highlighting the CAO gene’s role in shade tolerance [36]. Wang et al. (2015) elucidated the regulatory role of the *CarbcL* gene in modulating pepper fruit coloration under LL conditions [15]. Zhao et al. (2022) identified a major QTL, *qSAR1*, and key regulators such as *PIF7* associated with the inactive shade avoidance syndrome (iSAS) phenotype in the high-density planting-tolerant soybean cultivar JiDou 17, shedding light on soybean’s shade tolerance [37]. Su et al. (2023) identified 63 shade tolerance index genes, including *GmDREB2*, *GmNAC81*, and *GmWRKY27*, providing promising targets for breeding analyses [38]. Sahu et al. (2023) characterized morphological differences between LL-sensitive (IR-64) and LL-tolerant (Swarnaprabha) rice cultivars, offering genomic resources for isolating light-responsive photoreceptors in rice [39]. Despite these advancements, research gaps remain in understanding LL stress tolerance in rice. Specific QTLs and candidate genes governing grain yields under LL conditions require further exploration. Additional research is needed to validate identified candidate genes across diverse backgrounds and environments. Understanding these genes’ functions and interactions under LL stress is crucial for developing breeding strategies that enhance LL resilience in rice. Our study aimed to identify QTLs and candidate genes associated with grain yields and related traits under low-light (LL) stress using a recombinant inbred line (RIL) population derived from a cross between the LL-tolerant variety Swarnaprabha and the LL-susceptible variety IR8. Specifically, the objective was to discover QTLs and candidate genes linked to ten grain yield and related traits under both low-light (LL) and normal-light (NL) conditions.

## 2. Materials and Methods

### 2.1. Plant Materials

The low-light-tolerant variety, Swarnaprabha (PTB 43), and the susceptible high-yielding variety, IR8, were used as parents to develop a recombinant inbred line (RIL) population. Swarnaprabha is an early-maturing and high-yielding rice variety [29,40]. The RIL population was developed through the single-seed descent method during 2016–2021, and used to identify QTLs and candidate genes associated with grain yield and related traits under low-light stress conditions.

### 2.2. Phenotypic Evaluation of RIL Mapping Population for Grain Yield and Related Traits Under Low-Light Stress Conditions

One hundred and eighty-eight RILs along with their parents, Swarnaprabha and IR8, were evaluated for grain yield and nine related traits under low- and normal-light conditions following the alpha lattice design with two replications during the *Kharif* seasons of 2019 (F_8_) and 2021 (F_10_). Seeds from each RIL and parent were sown in lines in a raised bed during June. Twenty-five- to twenty-eight-day-old seedlings were transplanted in the experimental plots of the ICAR Central Rice Research Institute (CRRI), Cuttack, Odisha, India (20.4625° N, 85.8830° E), with spacing distances of 20 cm between rows and 15 cm between plants. Low-light stress was imposed after seven days of transplantation up to maturity by using agro-shade nets matted on a wooden frame with 25% interception of the photosynthetically active radiation, while no agro-shade net was used for the normal-light (NL) conditions. Fertilizer doses of 80 kg N_2_, 40 kg P_2_O_5_, and 40 kg K_2_O were applied, with 50% N_2_ and 100% P_2_O_5_ and K_2_O applied as basal doses during the final field preparation. The rest of the nitrogen fertilizer was split into two equal parts, with one half applied during tillering and the other half during the flowering stages. Standard agronomic practices and plant protection measures were followed for normal crop growth. Data were recorded on 10 traits, including the days to 50% flowering (DFF), plant height (PH), number of tillers per plant (TN), number of panicles per plant (PN), number of fertile grains per panicle (GN), number of spikelets per panicle (SN), spikelet fertility percentage (SFP), panicle weight (PW), 1000 grain weight (TGW), and grain yield per plant (GY), under both low and control (normal)-light conditions. Data were generated for five plants with two replications in each RIL, and mean values were used for the subsequent analysis.

### 2.3. Genotyping of Parents and RIL Mapping Population

High-quality genomic DNA was extracted from the young leaves of the parents and 188 RILs following the CTAB method used by Murray and Thompson [41]. The DNA quality and quantity were assessed using a NanoDrop spectrophotometer (Eppendorf Biospectrometer, Eppendorf AG, Germany) and 1.0% agarose gel electrophoresis. A total of 1407 markers, comprising 1183 rice microsatellite (RM) and 223 single-nucleotide polymorphic (SNP) markers evenly distributed across 12 rice chromosomes, were used to identify polymorphic markers between the parents, Swarnaprabha, and IR8. The RM primer sequences were obtained from the Gramene database. The SNP sequences were obtained from the OryzaSNP@MSU database, and MassARRAY Assay Design Suite (ADS) software version 4 was used to design the SNP primers. Twelve assay panels were designed, and polymorphism was surveyed using the AgenaMassArrayAnalyzer4 (MA4) genotyping system. Subsequently, 75 polymorphic SSR (simple sequence repeat) and 48 polymorphic SNP markers (Supplementary Tables S1 and S2) were utilized to genotype the 188 RILs along with the parents.

### 2.4. Construction of Linkage Map and Identification of QTLs

Genotypic data from 188 RILs generated by 75 SSR and 48 SNP polymorphic markers were used to construct a linkage map using the integrated QTL IciMapping software Version 4.2 [42]. The Kosambi mapping function was used to convert recombination frequencies to genetic distances in centimorgans (cM) [43]. The same software was used to identify QTLs associated with grain yield and related traits under low and normal conditions using the inclusive composite interval mapping (ICIM) method. ICIM is a more efficient background control method than composite interval mapping (CIM). It avoids the possible increase in sampling variance and the complicated background marker selection process in CIM [44,45]. A one-dimensional scan of the whole genome was carried out with a threshold LOD score of 2.5 to identify significant QTLs [46]. The identified QTLs were named according to McCouch et al. [47].

### 2.5. In Silico Analysis to Identify Candidate GENES Associated with QTLs

The genes were retrieved from the ‘search and retrieval’ tool of the RAP-DB database (https://rapdb.dna.affrc.go.jp/ (accessed on 1 April 2025)) for rice. The genes flanking 500 kb regions of the three QTL hotspot regions were identified chromosome-wise. Further, gene annotations in the RAP-DB database of the candidate genes were searched for keywords such as grain-yield-related traits, starch and amylose contents, photosynthesis-related traits, and light sensitivity traits for the selection of the candidate genes (Supplementary Table S4).

### 2.6. Expression Analysis of Candidate Genes

Primers were designed for the selected 10 candidate genes with Tm 58 °C to 62 °C and a length of 19-mer to 22-mer using the Primer Blast software at the NCBI site (http://www.ncbi.nlm.nih.gov/tools/primer-blast/, accessed on 4 July 2025) (Supplementary Table S5). The expression of these 10 genes was analyzed using qRT-PCR [29]. Leaves from low-light-treated and untreated plants of Swarnaprabha and IR8 were sampled 0, 1, 5, and 10 days after anthesis; frozen into liquid nitrogen; and stored at −80 °C until use. The total RNA was isolated from the collected samples using the RNEasy Plant Mini Kit (Qiagen, Redwood City, CA, USA) following the manufacturer’s protocol. The quality and quantity of the RNA isolated from the individual samples were checked on an agarose gel and a nanodrop spectrophotometer. The Quanti-Tect Reverse Transcription Kit (Qiagen, Redwood City, CA, USA) was used for the conversion of the total isolated RNA to cDNA following the protocol outlined in the kit’s manual. The expression of a gene was studied via qRT-PCR, taking the cDNA as a template and using SYBR green (Agilent, Santa Clara, CA, USA). The required amount of SYBR green, cDNA template, and primers for a gene were mixed in a final volume of 20 µL, and the PCR was run in Real-Time PCR system (Bio-Rad CFX, Hercules, CA, USA). Rice actin was used as an internal control. The relative level of the template of the individual gene in the leaves was quantified using the ^ΔΔCT^method [48], and the result was expressed as a fold change in the expression of the samples under low-light stress compared to the normal-light conditions.

### 2.7. Pathway and Network Analysis of Selected Candidate Genes

The identified candidate genes were then fed into RiceFREND (Rice Functionally Related Gene Expression Network Database) for the expression network analysis, and the interactions were identified [49]. The network was then visualized using Cytoscape v. 3.10.1 [50]. The hub genes were then identified using the cytoHubba v0.1 plugin of Cytoscape, which uses the maximal clique centrality (MCC) algorithm to get the top genes. The MCODE plugin of Cytoscape was also used to identify the densely connected genes among the interactions. The pathway analysis was carried out using the KEGG pathway database (Kyoto Encyclopedia on Genes and Genomes) [51].

### 2.8. Statistical Analysis

The descriptive statistical analysis was carried out for standard statistical measures, such as the mean, range, standard deviation (SD), standard error (SE), coefficient of variation (CV%), skewness, and kurtosis in each of the 10 phenotypic traits of the RILs. The frequency distribution graphs were plotted for all 10 traits and calculated using the statistical software package SPSS (IBM SPSS Version 23.0). The statistical analysis of the mean values of five randomly selected plants from each of the two replications for 188 RILs along with the parents (both under LL and NL conditions) for the years 2019 and 2021 (*Kharif*) was carried out on individual traits. The mean values for all traits were analyzed for their variance following the alpha lattice design. An analysis of variance was carried out using ICIMapping version 4.2 [42] along with the Tukey–Kramer method through Microsoft Excel 2019. Using the Tukey–Kramer method, the minimum significant difference (MSD) was calculated for each pair of means. This method depends on the sample size in each group, the average variation within the groups, and the total number of groups. It can be used to find means that are significantly different from each other. The significance was tested by referring to the table given by Fisher [52]. The phenotypic correlations and principal component analysis (PCA) were estimated using the XLSTAT-23 software Addinsoft [53].

## 3. Results

### 3.1. Phenotypic Variations and Correlations Among Traits in the RIL Population

**Figure 1:**
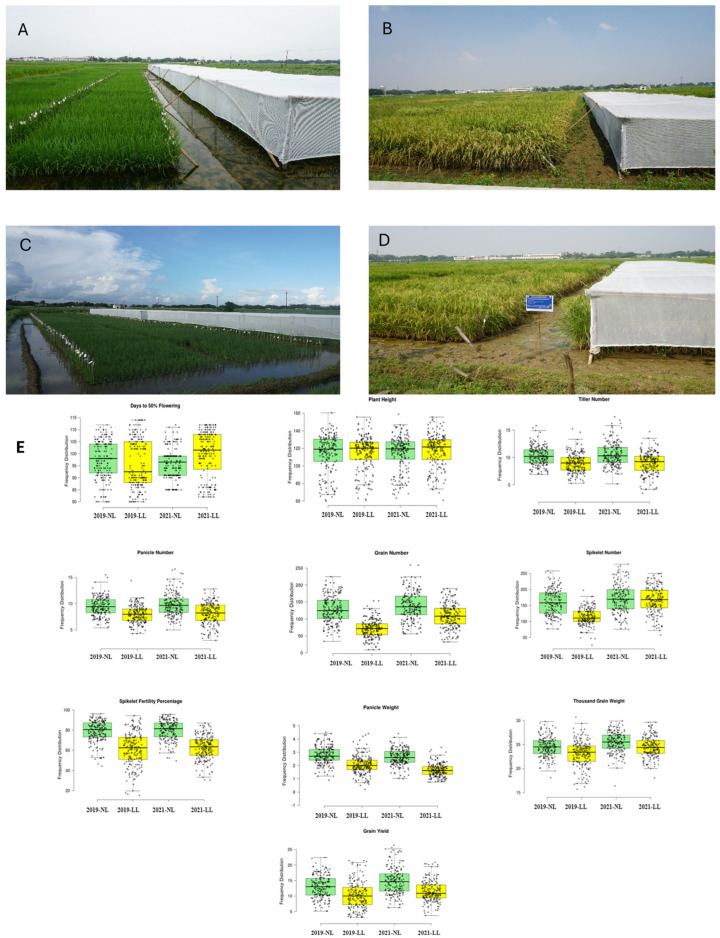
Evaluation of RIL mapping population developed from the cross between the low-light-tolerant variety Swarnaprabha and the susceptible variety IR8 at the vegetative stage (**A**,**C**) and reproductive stage (**B**,**D**) grown under low-light and normal (control)-light conditions during the *Kharif* seasons of 2019 (**A**,**B**) and 2021 (**D**). Box plots of grain yield and nine related traits describing frequency distributions under low-light (LL) and normal (control)-light (NL) conditions during *Kharif* seasons of 2019 and 2021. Traits are mentioned above the figure (**E**).

Phenotypic variations within the RIL population were analyzed and frequency distribution box plots for 10 grain yield and related traits under LL and NL conditions were generated (Figure 1). Descriptive statistics, including coefficients of variation (CV%), skewness, kurtosis, and range, were calculated for 10 yield-related traits across both *Kharif* seasons of 2019 and 2021 under LL and NL conditions. A broad range of CV% values was observed. These values varied from 10.57% to 41.38% under LL and 8.11% to 30.57% under NL conditions in *Kharif* 2019, while they ranged from 8.58% to 32.25% under LL and 6.49% to 30.26% under NL conditions in *Kharif* 2021. These variations indicated that there was a significant influence of environmental conditions on the expression of 10 grain yield and related traits. Significant differences between the LL and NL conditions were observed for all traits (*p* ≤ 0.05) except plant height in *Kharif* 2019 (*p* = 0.25). The distribution of the plant height, spikelet number, and spikelet fertility percentage values under both LL and NL conditions was found to be platykurtic and negatively skewed across seasons (Supplementary Table S3).

To further dissect the variance among traits, an analysis of variance (ANOVA) was performed across seasons using the best linear unbiased predictors (BLUP). The analysis revealed significant variations between environments and genotypes (RILs) for all ten traits (*p* ≤ 0.05). Similarly, all traits except plant height (0.99) displayed significant genotype–environment interactions (*p* ≤ 0.05), indicating the influence of both genetic and non-genetic factors. The proportions of variance explained (R^2^) ranged from 65.07% to 91.59%, emphasizing the substantial trait variations observed across environments. The Bartlett test for homogeneity was significant for all ten traits (*p* ≤ 0.05), reinforcing the impacts of the genotype, environment, and their interactions on trait variability (Supplementary Table S4).

**Figure 2:**
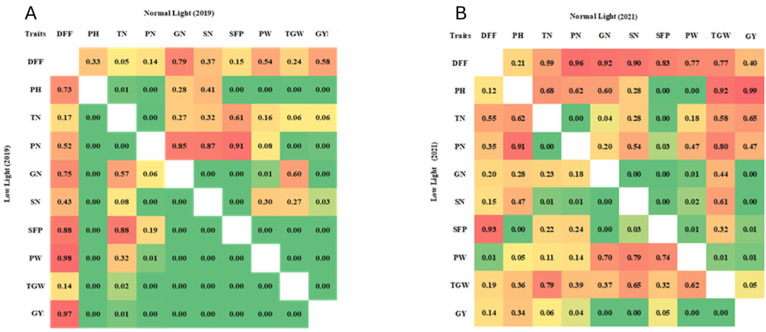
Corrplot describing the *p*-values of Pearson’s correlation coefficients among grain yield and related traits under low-light and normal (control)-light conditions during *Kharif* season 2019 (**A**) and *Kharif* season 2021 (**B**). The values in the light yellow-green, light green, and green color boxes are significant at the *p* values of ≤0.05, ≤0.01, and ≤0.001 levels, respectively. DFF: days to 50% flowering; PH: plant height (cm); TN: tiller number; PN: panicle number; GN: grain number per panicle; SN: spikelet number per panicle; SFP: spikelet fertility percentage; PW: panicle weight (g); TGW: thousand grain weight (g); GY: grain yield per plant (g).

The Pearson’s correlation coefficient analysis showed significant positive correlations (*p*-value) between the grain yield (GY) and PH (0.01), TN (0.001), PN (0.001), GN (0.001), SN (0.001), SFP (0.001), PW (0.001), and TGW (0.001) in *Kharif* 2019, while PN (0.04), GN (0.001), SN (0.001), SFP (0.05), PW (0.001), and TGW (0.001) showed significant positive correlations in *Kharif* 2021 under the LL conditions. Similarly, GN, PN, SFP, PW, and TGW showed significant correlations with GY (*p* ≤ 0.05) under the NL conditions in both seasons. Positive correlations were observed between SN and GN (0.001, 0.05), as well as SFP and GY (0.001, 0.01), respectively, in the *Kharif* seasons of 2019 and 2021 under both conditions (Figure 2). The correlations suggested that these traits are closely linked and co-regulated under varying light conditions, making them useful for breeding programs for improving yields under LL stress.

### 3.2. Genotyping and Linkage Map Construction

A set of 1407 markers, comprising 1183 simple sequence repeat (SSR) markers and 224 single-nucleotide polymorphism (SNP) markers, were used to survey polymorphism between the parental genotypes Swarnaprabha and IR8. In total, 123 markers (8.74%) were found to be polymorphic between the parents and subsequently used to genotype 188 RILs along with the parents (Table 1). A linkage map was constructed using the genotypic data generated from the 188 RILs, which resulted in thirteen linkage groups, covering a total distance of 2397.17 cM across the genome, with an average inter-marker distance of 22.61 cM (Supplementary Table S5). Chromosomes 1 and 8 exhibited two linkage groups, while the remaining chromosomes had a single linkage group. Our analysis of the physical and genetic distance ratios between consecutive markers revealed a non-linear relationship and varying recombination rates along chromosome lengths and across different chromosomes. Recombination hotspots were found to be dispersed throughout the genome.

### 3.3. Composite Interval Mapping and Identification of QTLs

**Figure 3:**
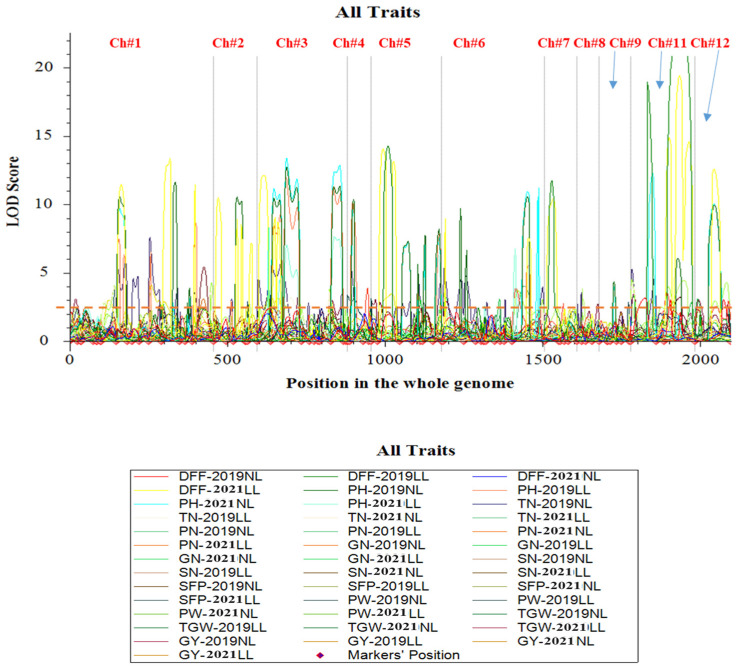
Whole-genome scanning for the identification of QTLs associated with grain yield and nine related traits under low-light (LL) and normal (control)-light (LL) conditions during the *Kharif* seasons of 2019 and 2021. The *X*-axis shows the positions of markers on chromosomes based on the cM position, while the *Y*-axis shows the LOD score, the red dashed line shows the minimum LOD value of 2.5.

**Figure 4:**
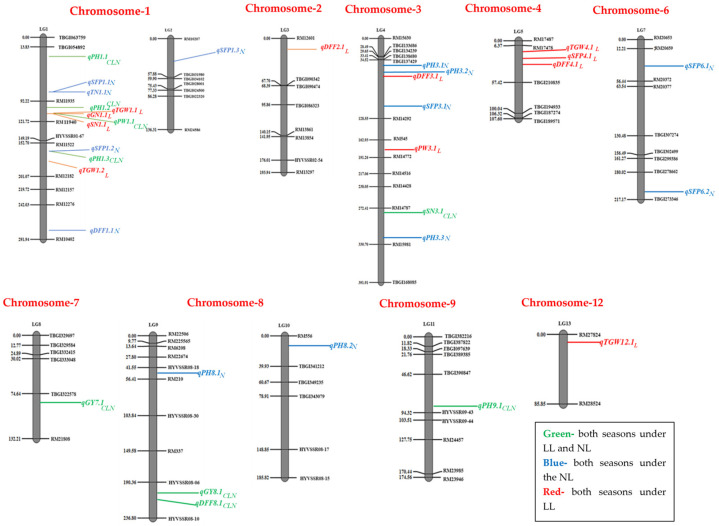
Linkage map showing locations of markers and QTLs associated with grain yield and related traits under low-light (LL) and normal (control)-light (NL) conditions in both *Kharif* seasons of 2019 and 2021. DFF: days to 50% flowering; PH: plant height (cm); TN: tiller number; PN: panicle number; GN: grain number per panicle; SN: spikelet number per panicle; SFP: spikelet fertility percentage; PW: panicle weight (g); TGW: thousand grain weight (g); GY: grain yield per plant (g).

A total of 67 QTLs for ten traits were identified in two *Kharif* seasons (2019 and 2021), both under low-light (LL) and control (normal)-light (NL) conditions (Supplementary Table S6, Figure 3). Under LL conditions, 11 QTLs (*qDFF5.1_L_*, *qDFF7.1_L_*, *qGN9.1_L_*, *qSFP8.4_L_*, *qPW1.2_L_*, *qPW1.3_L_*, *qPW9.1_L_*, *qPW9.2_L_*, *qGY1.1_L_*, and *qGY1.2_L_*) were found for six traits in *Kharif* 2019, while six QTLs (*qDFF5.2_L_*, *qDFF8.2_L_*, *qTN8.2_L_*, *qPN4.2_L_*, *qPW3.2_L_*, and *qGY3.1_L_*) were identified for five traits in *Kharif* 2021. Under NL conditions, 10 QTLs (*qPH8.3_N_*, *qTN8.1_N_*, *qGN3.1_N_*, *qSN3.2_N_*, *qSFP8.1_N_*, *qSFP8.2_N_*, *qSFP8.3_N_*, *qTGW8.1_N_*, *qGY3.2_N_*, and *qGY7.2_N_*) were identified for eight traits in *Kharif* 2019, while seven QTLs (*qTN8.3_N_*, *qPN3.1_N_*, *qPN3.2_N_*, *qPN7.1_N_*, *qPN8.1_N_,* and *qPW4.1_N_*) were identified for three traits in *Kharif* 2021. Nine QTLs (*qDFF8.1_CLN_*, *qPH1.1_CLN_*, *qPH1.2_CLN_*, *qPH1.3_CLN_*, *qPH9.1_CLN_*, *qSN3.1_CLN_*, *qPW1.1_CLN_*, *qY7.1_CLN_*, and *qGY8.1_CLN_*) were identified under both LL and NL conditions in both *Kharif* seasons. Out of 67 QTLs, 21 QTLs (11 under LL and 10 under NL conditions) were found only in *Kharif* 2019, while 13 QTLs (6 under LL and 7 under NL conditions) were found only in *Kharif* 2021. These 34 QTLs were inconsistent and lacked stability, as they were detected exclusively in one of the two *Kharif* seasons and were excluded from further consideration. The remaining 33 QTLs, which were detected in both *Kharif* seasons, were selected for subsequent investigation. Detailed information about the location, phenotypic variance, and additive effect of the QTLs detected for the yield and yield-related traits is given in Table 2. In total, 11 QTLs were identified for six traits (DFF, GN, SN, SFP, PW, and TGW) only under LL conditions, while 13 QTLs were identified for four traits (DFF, PH, TN, and SFP) only under NL conditions in both the *Kharif* seasons (2019 and 2021). Nine QTLs were detected for five traits (DFF, PH, SN, PW, and GY) under both light conditions (LL and NL) in both years (Supplementary Table S6). The numbers of stable QTLs detected for each trait varied from 1 to 9, with the phenotypic variance levels ranging from 4.16% to 18.78% and the additive effects ranging from −1.03 to 18.77 in both seasons among the grain yield and related traits under low- and normal-light conditions. The locations of QTLs on the linkage map are presented in Figure 4. In total, 24 QTLs were contributed by Swarnaprabha (eight only under LL, eight only under NL, and eight under both conditions), while nine QTLs were contributed by IR8 (three only under LL, five only under NL, and only one under both conditions).

### 3.4. Novel QTLs

Among the 33 stable QTLs identified in this study, 11 QTLs (*qDFF2.1_L_*, *qDFF3.1_L_, qDFF4.1_L_*, *qGN1.1_L_*, *qSN1.1_L_*, *qSFP4.1_L_*, *qPW3.1_L_*, *qTGW1.1_L_*, *qTGW1.2_L_*, *qTGW4.1_L_*, and *qTGW12.1_L_*) were identified for six traits exclusive to low-light (LL) conditions, while nine QTLs (*qDFF8.1_CLN_*, *qPH1.1_CLN_*, *qPH1.2_CLN_*, *qPH1.3_CLN_*, *qPH9.1_CLN_*, *qSN3.1_CLN_*, *qPW1.1_CLN_*, *qGY7.1_CLN_*, and *qGY8.1_CLN_*) were detected for five traits under both LL and normal-light (NL) conditions. These QTLs are novel, since they were not identified before under low-light conditions (Table 2). Out of the twenty novel QTLs, Swarnaprabha, known for its low-light tolerance, contributed sixteen, while IR8 contributed four. The traits with the highest numbers of novel QTLs were the days to 50% flowering (DFF), plant height (PH), and thousand grain weight (TGW) with four each, followed by the spikelet number (SN), panicle weight (PW), and grain yield (GY) with two each and the grain number (GN) and spikelets per panicle (SFP) with one each. These novel QTLs were distributed across all chromosomes except for 5, 6, 10, and 11. The highest number of novel QTLs (i.e., 8) was detected on chromosome 1, followed by chromosomes 3 and 4 with 3 each. Two novel QTLs, *qDFF2.1_L_* and *qDFF4.1_L_*, exhibited expression across two seasons for the days to 50% flowering, with the LOD values ranging from 10.32 to 18.39 and explaining 10.21–11.32% of the phenotypic variation. Two novel QTLs, *qGY7.1_CLN_* and *qGY8.1_CLN_*, were identified for grain yield under both LL and NL conditions, with the phenotypic variance levels explained by these QTLs ranging from 11.27% to 18.78% and the LOD values ranging from 2.89 to 4.18.

### 3.5. QTL Hotspots

Three QTL hotspots (clusters), one each on chromosomes 1, 4, and 8, were identified for eight traits—the days to 50% flowering, plant height, grain number per panicle, spikelet number per panicle, spikelet fertility percentage, panicle weight, 1000 grain weight, and grain yield (Table 3). QTL hot spot I, flanked by RM11935-RM11940, harbored QTLs for five traits—the plant height *(qPH1.2_CLN_*), grain number per panicle (*qGN1.1_L_*), spikelet number per panicle (*qSN1.11_L_*), panicle weight (*qPW1.1_CLN_*), and thousand grain weight per plant (*qTGW1.1_L_)*—in a window size of 0.1 Mb. Swarnaprabha contributed all five QTLs. Three QTLs controlling the days to 50% flowering (*qDFF4.1_L_*), spikelet fertility percentage *(qSFP4.1_L_*), and thousand grain weight per plant (*qTGW4.1_L_*) with a window size of 3.2 Mb under LL conditions are included in hotspot II, flanked by RM17478-TBGI210835. Hotspot III, flanked by HYVSSR8-06-HYVSSR8-10, harbored two QTLs controlling traits such as the days to 50% flowering (*qDFF8.1_CLN_*) and grain yield (*qGY8.1_CLN_*), with a window size of 0.6 Mb under both LL and NL conditions.

### 3.6. In Silico Analysis for the Identification of Candidate Genes in QTL Hotspot Genomic Regions

A total of 239, 516, and 171 genes were identified in hotspot region I (37.7 to 37.8 Mb, hotspot II (27.6 to 30.8 Mb), and hotspot III (4.6 Mb to 5.2 Mb position), respectively (Table 3). While analyzing the protein sequences and gene and trait ontologies of these genes, we found that hotspots I, II, and III contain 17, 1, and 2 candidate genes, respectively, which are responsible for photosynthesis, the starch and amylose contents, grain-yield-related traits, and temperature-responsive traits (Supplementary Table S7). Among these 20 candidate genes, eight from chromosome 1, one from chromosome 4, and one from chromosome 8 were selected for an expression study.

### 3.7. Expression Analysis of Candidate Genes

**Figure 5:**
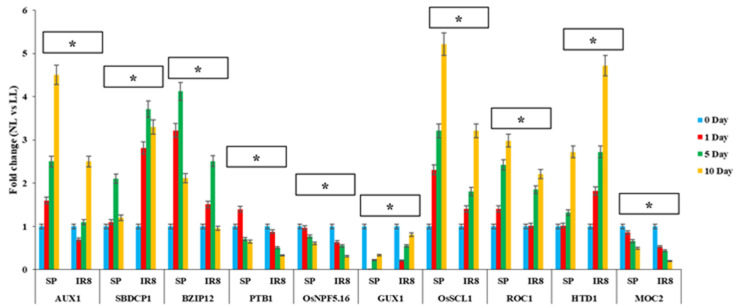
Expression patterns of 10 candidate genes associated with grain yield and related traits in the leaves of Swarnaprabha (SP) (T) and IR8 (S), sampled 0, 1, 5, and 10 days after anthesis under low-light (LL) and normal (control)-light (NL conditions through qRT-PCR. Each gene was differentially expressed with respect to the control and amplified with gene-specific primers designed using Primer Blast. Actin was taken as an internal positive control. Each data point is the average of three replicates, and the error bars represent the standard error (SE). Asterisks indicate significant differences determined by the *t*-test (* *p* < 0.05). Candidate genes are presented at the bottom of the graph.

To validate the involvement of the 10 identified candidate genes in the QTL hot spot regions (*qPH1.2_CLN_*, *qGN1.1_L_*, *qSN1.1_L_*, *qPW1.1_CLN_*, *qTGW1.1_L_*, *qDFF4.1_L_*, *qSFP4.1_L_*, *qTGW4.1_L_*, *qDFF8.1_CLN_*, and *qGY8.1_CLN_*) associated with different traits under LL conditions, comparative expression studies of these genes were carried out at the reproductive stage of Swarnaprabha and IR8 on the days 0, 1, 5, and 10 of the low-light treatment (Figure 5, Supplementary Table S8). These candidate genes are related to *OsAUX1* (Os01g0856500), starch binding domain-containing protein 1, *OsSBDCP1* (Os01g0856900), b-ZIP transcription factor 12, *BZIP12* (Os01g0866400), polypyrimidine-tract-binding protein 1, *OsPTB1* (Os01g0867800), nitrate transporter 1/peptide transporter 5.16, *OsNPF5.16* (Os01g0872500), plant-glycogenin-like starch initiation protein A1, *OsGUX1* (Os01g0880200), chitin-inducible gibberellin-responsive protein, plant height 1, *OsSCL1* (Os01g0881500), rice outmost cell-specific gene 1, *OsROC1* (Os08g0187500), high-tillering dwarf1, *OsHTD1* (Os04g0550600), MONOCULM 2, cytosolic fructose-1, 6-bisphosphatase 1, and *OsMOC2* (Os01g0866400), and were previously reported for grain yield traits, temperature response traits, auxin and gibberellic acid sensitivity, starch amylose contents, and several morphological and physiological traits under different abiotic stress and normal conditions but not under low-light stress conditions. The expression patterns of the *OsAUX1*, *OsSCL1*, *ROC1*, and *HTD1* genes exhibited significant upregulation under low-light stress up to the 10th day, while the *SBDCP1* and *BZIP12* genes displayed an upregulation pattern until the 5th day, followed by a decrease. Notably, the expression values were consistently higher in Swarnaprabha compared to IR8. However, the *PTB1*, *OsNPF5.16*, and *MOC2* genes demonstrated downregulation under low-light stress, with higher expression levels observed in Swarnaprabha. An exception was observed in the *OsAUX1* gene, where down regulation occurred under low-light stress but the expression was higher in IR8 than in Swarnaprabha.

### 3.8. Pathway and Network Analysis of Hub Genes

The pathway and network analysis of ten candidate genes led to the identification of three genes (*OsAUX1*, *OsSBDCP1*, and *OsNPF5.16*) that significantly co-expressed with each other and served as central hubs in key regulatory networks involved in hormone signaling, starch metabolism, and nitrogen assimilation (Supplementary Figure S1). The candidate gene *OsSBDCP1* (Os01g0856900) is a starch-binding protein controlling the amylose content and amylopectin content (Supplementary Figure S2). Apart from this gene, two other important candidate genes, *OsAUX1* (Os01g0856500) and *OsNPF5.16* (Os01g0872500), were analyzed in detail with their respective pathways, such as the plant hormone signal transduction pathway (Supplementary Figure S3) and the nitrogen metabolism pathway (Supplementary Figure S4).

## 4. Discussion

Low light is one of the major abiotic stresses in the rainy season. It alters the chlorophyll content and composition, thereby hampering the photosynthetic activity and impeding growth and biomass accumulation [54]. Particularly during crucial growth stages such as tillering, flowering, and grain filling, insufficient light availability adversely affects the tiller and panicle development, pollen viability, grain filling, and grain quality, ultimately leading to reduced grain yields [55]. Studies have indicated reductions in total chlorophyll content while increasing the chlorophyll a/b ratio under low-light conditions [19]. Moreover, rice plants under low-light stress exhibit increased vulnerability to various biotic and abiotic stresses, including pest infestations, diseases, drought, and nutrient deficiencies. This compromised physiological state weakens the plant’s resilience against stressors, further emphasizing the impact of low-light conditions [56].

To overcome low-light stress in rice, various strategies have been employed, including the utilization of marker-assisted selection (MAS) programs. The successful implementation of MAS relies on comprehensive knowledge of the underlying genetic elements within genomic regions on chromosomes that influence target traits and their interactions across diverse environments [57]. To generate the requisite information, mapping target traits such as the grain yield and related QTLs/genes is pivotal. Immortal populations such as recombinant inbred lines (RILs), doubled haploids (DH), and backcross inbred lines (BILs) offer valuable resources for the identification of QTLs and genes. RILs, derived from multiple meiotic cycles, surpass DH populations by providing a desired structure and exhibiting twice the observed recombination between closely linked markers [58]. Moreover, RILs offer the advantage of indefinite maintenance and unlimited seed supplies for repeated experiments over multiple years and locations, facilitating the estimation of QTL–environment interactions [59]. In the present study, an RIL was developed from the cross between the low-light-tolerant rice variety Swarnaprabha and the low-light-susceptible variety IR8 and was used to identify specific genomic regions (QTLs) linked to grain yield and related traits under low-light and normal conditions. 

### 4.1. Phenotypic Diversity Among Parents and RILs

Our study revealed substantial genetic diversity among both the parents and the RILs across all ten traits. Wide variation was observed under both LL and NL conditions during the *Kharif* seasons of 2019 and 2021, with the exception of the plant height in 2019. Significant differences in trait ranges were evident in both years, with the number of grains per panicle showing the greatest variability, followed by the spikelet number per panicle and plant height. In contrast, the days to 50% flowering, panicle weight, and thousand grain weight exhibited the narrowest ranges across both light regimes and seasons. The spikelet number per panicle showed a high mean and high variability; hence, it is best to select plants based on the same traits. Similar reports were observed by others [60,61,62,63,64,65]. The analysis of skewness and kurtosis indicated that the population showed a normal distribution for all the traits except for the plant height (Supplementary Table S3), whereas platykurtic and negatively skewed distributions were recorded for the plant height, spikelet number, and spikelet fertility percentage under both LL and NL for both seasons, indicating that these traits are governed by major QTLs. The majority of them have positive effects, displaying dominant and dominant-based duplicate epistasis inheritance. Hence, mild selection is expected to result in rapid genetic gain for these traits. These results agree with the findings for the test weight, grain length, and grain breadth, as well as for the plant height [66,67].

Genetic and environmental variations were studied for 10 traits across two growing seasons. The genotype-by-environment interaction was significant for all traits except the plant height (Supplementary Table S4). Khan et al. [68] and Khan et al. [69] reported similar findings, who investigated the genetic and environmental variations of the grain yield and its components in rice under different environmental conditions. They found that the proportion of variance explained by the latest research on the genotype–environment interaction (GEI) has shown that it is a major source of variation in quantitative traits, such as the grain yield. GEI occurs when the phenotypic expression of a trait is affected by the interaction between the genotype of the plant and the environment in which it is grown. This can make it difficult to predict how a genotype will perform in a new environment [70].

### 4.2. Correlations Among Grain Yield and Related Traits

The positive correlations between the grain yield (GY) and other traits indicate shared genetic control, suggesting that QTLs or genes influencing GY may also affect these related traits. Similarly, strong positive associations were observed between the spikelet number (SN) and grain number (GN), as well as between the spikelet fertility percentage (SFP) and GY, further supporting the presence of underlying genetic relationships among these traits. Similarly, the positive correlation coefficients were obtained by Babaret al. [71] for TN; Krishnan et al. [72] for Pl; and Kumar et al. [73] for DFF, TN, GN, TGW, and SFP. Positive correlation coefficients were also obtained by Mohanty et al. [74]. Other than GY, PH and TGW showed significant positive correlations with TN, PN, GN, SN, SFP, and PW under LL conditions for *Kharif* 2019, while SN showed significant positive correlations with TN, PN, GN, and SFP under LL conditions for both seasons. Similar reports have been found by [75,76]. According to Agalya et al. [76], there are strong positive connections between the panicle weight, number of filled grains per panicle, 1000 grain weight, and single plant yield. Biswas et al. [75] found a strong positive correlation between the plant height, 100 grain weight, grain breadth, leaf breadth, and crop yield. This suggests that selecting plants with these characteristics could be a good strategy for increasing yields.

### 4.3. Identification of QTLs Associated with Grain Yield and Related Traits Under Low-Light and Normal-Light Conditions

Of the 1407 markers we screened, 123 (8.74%) were polymorphic between Swarnaprabha and IR8 (Table 1). This low rate of polymorphism was because both parents are from Indian backgrounds. This is consistent with a previous study by Yang et al. [77], who found that the average polymorphism rate between two rice varieties was 8.5%. Our study constructed a linkage map length of 2297.64 cM, with 13 linkage groups and an average distance of 22.75 cM between markers. Similarly, a linkage map of 75 SSR markers was constructed in an RIL population for identifying QTLs for BPH resistance, with a total map length of 1251.78 cM and an average interval of 17.30 cM [78]. Verma et al. [79] constructed a linkage map of 89 SSR markers covering a total of 1628.7 cM of the rice genome, with an average marker density of 18.3 cM. An analysis of the ratios of physical and genetic distances revealed that the recombination rates varied along chromosome length and from chromosome to chromosome due to the unequal physical and genetic distances between markers [80].

A total of 67 QTLs were identified across the *Kharif* seasons of 2019 and 2021 under both LL and NL conditions, with 33 detected in both years. Specifically, 11 QTLs were unique to LL, 13 to NL, and 9 were present under both light conditions. Notably, 20 QTLs (including *qDFF2.1_L_*, *qGN1.1_L_*, *qGY7.1_CLN_*, and others) were novel, as none had been previously reported for yield and related traits under low-light stress, based on GRAMENE, Q-Taro, and Oryzabase databases. Previous studies have identified a few novel QTLs for yield and related traits in rice under normal and different environmental conditions. Shekhar et al. [81] identified seven novel QTLs associated with ethylene production (*qETH1.1*, *qETH1.2*, *qETH3.1*, *qETH4.1*, *qETH4.2*, *qETH6.1*, and *qETH6.2*) for poor grain filling of basal spikelets in dense panicle rice under normal-light conditions. Similarly, Malik et al. [82] identified five novel QTLs for grain yield and its associated traits using an RIL population from the cross of Sonasal and Pusa Basmati 1121. Li et al. [83] identified a novel QTL, *qRSL1-2,* related to the RSL (relative shoot length) for salt tolerance at the bud burst stage in rice. Donde et al. [84] identified thirty novel QTLs for traits such as the tiller number, panicle length, flag leaf size, total grains, thousand grain weight, fertile grains, seed length-to-breadth ratio, plant height, days to 50% flowering, and grain yield in rice. Similarly, a study with 48 rice genotypes under low-light conditions identified six markers—HvSSR02-44 (biological yield); HvSSR02-52, HvSSR06-56, HvSSR06-69, and HvSSR09-45 (spikelet fertility); and HvSSR02-54 (grain yield)—associated with yield-related traits [33]. HvSSR02-54, positioned at 21.8 Mb on chromosome 2, exhibited a robust correlation with the grain yield under low-light conditions. However, our investigation did not reveal any QTLs associated with HvSSR02-54 or the other five markers.

### 4.4. Identification of QTL Hotspots

The QTL hotspots provide valuable information for the optimization of breeding strategies. Focusing on these genomic regions enables breeders to design cultivars with enhanced agronomic traits. We identified three hotspot regions for QTLs associated with different traits. Ten QTLs were identified in three hotspot regions on chromosomes 1, 4, and 8 with marker intervals RM11935-RM11940, RM17478-TBGI210835, and HYVSSR8-06-HYVSSR8-10, respectively. Hotspot I on chromosome 1 could be targeted to develop varieties with specific plant height *(qPH1.2_CLN_*), grain number per panicle (*qGN1.1_L_*), spikelet number per panicle (*qSN1.1_L_*), panicle weight (*qPW1.1_CLN_*), and thousand grain weight per plant (*qTGW1.1_L_*) characteristics. Hotspot II on chromosome 4 presents an opportunity for breeders to develop varieties exhibiting specific days to 50% flowering (*qDFF4.1_L_*), spikelet fertility percentage *(qSFP4.1_L_*), and thousand grain weight per plant (*qTGW4.1_L_*) characteristics. Targeting Hotspot III on chromosome 8 is conducive to the development of varieties characterized by specific days to 50% flowering (*qDFF8.1_CLN_*) and grain yield (*qGY8.1_CLN_*) characteristics. Marathi et al. [85] identified fifteen QTL hotspots across chromosomes 1–4, 6–8, and 12, containing QTLs for key traits such as the plant height, panicle length, flag leaf attributes, spikelets per panicle, filled grains per panicle, and spikelet setting density. Sahu et al. [39] identified 5 QTL hotspots containing 37 QTLs influencing grain yield traits on chromosomes 3 and 4. Kulkarni et al. [86] identified a QTL hotspot on chromosome 3 for the total grain yield/plant (*qYLD3-1)* and panicle length (*qPL3-1*) in a recombinant inbred line (RIL) population derived from the popular rice hybrid KRH-2.

### 4.5. Identification and Expression Analysis of Candidate Genes Associated with QTL Hotspots

Twenty candidate genes related to grain yield traits were identified in the three QTL hotspot regions (17 in hotspot I on chromosome 1, two in hotspot II on chromosome 4, and one in hotspot III on chromosome 8). We selected 10 candidate genes (*OsAUX1*, *OsSBDCP1*, *OsbZIP12*, *OsPTB1*, *OsNPF5.16*, *OsGUX1*, *OsSCL1*, *OsMOC2*, *HTD1*, and *OsROC1*) for an expression analysis through RT-PCR and qRT-PCR in the parents, Swarnaprabha (low-light-tolerant) and IR8 (low-light-susceptible). These genes are known for their involvement in various traits, such as the grain yield, auxin and gibberellic acid sensitivity, starch amylose content, and morphological and physiological traits under abiotic stress conditions. These genes were previously unexplored in the context of low-light stress in rice. *OsAUX1*, encoding an auxin influx transporter, emerged as a key player in rice plants’ low-light sensitivity. Auxin, a pivotal plant hormone, influences various growth and development processes, including photomorphogenesis. The study found that *OsAUX1* expression is regulated by light, with greater expression observed under low-light conditions. This implies that *OsAUX1* contributes to the adaptation of rice plants to low light, a hypothesis supported by increased expression in low-light conditions compared to highlight conditions [87]. The upregulation of *OsAUX1* in response to auxin and abiotic stress underscores its multifaceted role in plant adaptation. The gene ontology and trait ontology of candidate genes revealed that the tiller number, plant height, floral organ development trait, and days to heading are highly correlated with the *AUX1* gene. Similarly, *OsSBDCP1* is related to the starch and sucrose metabolism pathways. It represents the amylose content and amylopectin content of the starch-binding domain containing protein 1 with starch- and glycogen-forming properties. It was identified as a negative regulator of starch biosynthesis and exhibited higher expression under low-light conditions. This upregulation is postulated to slow down starch synthesis, aligning with the decreased grain filling rates observed under low light. Cakir et al. [88] emphasized the role of *SBDCP1* in inhibiting starch synthase IIIa, contributing to a better understanding of its impact on starch metabolism during grain filling under low light. The *MOC2* gene was downregulated under low-light stress but the expression was higher in tolerant than susceptible genotypes. The *MOC2* gene encodes a protein called cytosolic fructose-1,6-bisphosphatase 1 (*FBP1*). This enzyme is essential for sucrose biosynthesis, a key sugar molecule in plants. A mutation in *MOC2* disrupts *FBP1* function, likely leading to a shortage of sucrose and hindering tiller bud outgrowth [89]. According to Lee et al. [90], *OsMOC2* controls the conversion of sugar molecules for plant growth. When mutated, rice plants cannot make enough sucrose, leading to stunted growth. This suggests that rice relies more heavily on sucrose production than similar plants such as *Arabidopsis*.

*BZIP12*, a transcription factor, showed relative overexpression in Swarnaprabha compared to IR8 under low light. *BZIP12*′s binding to ABA-responsive elements (ABREs) during stress exposure suggests its involvement in ABA signaling, aiding plants in coping with adverse conditions [91]. According to Zhang et al. [92], the molecular mechanisms underlying the photoperiod or temperature control of the flowering time are regulated by the *bZIP* transcriptional factor, *O. sativa* ABA-responsive element binding factor 1 (*OsABF1*). The superior low-light stress resilience observed in Swarnaprabha, as indicated by increased *BZIP12* expression, hints at its potential role in enhancing stress tolerance. *PTBP1*, known for its role in grain yield regulation, exhibited differential downregulation under low-light conditions, with a significantly lower rate in IR8 compared to Swarnaprabha. This confirms studies demonstrating the importance of *PTBP1* in normal grain development in rice [93]. The observed differences in *PTBP1* expression may contribute to variations in grain yield under low-light stress between the two varieties. *OsNPF5.16*, a nitrate transporter gene with natural variation in its promoter sequence, is essential for rice growth and yields. *OsNPF5.16* was reduced under low light, which was relatively higher in Swarnaprabhathan in IR8. *Nitrate Transporter 1 (NRT1)* is a protein that helps rice plants absorb nitrate from the soil. As the photosynthetic capacity of the leaves is related to the nitrogen content, primarily because the proteins of the Calvin cycle and thylakoids represent the majority of leaf nitrogen, the rate of nitrogen absorption reduces under shade [94,95]. According to Wang et al. [96], *OsNPF5.16* plays a key role in rice growth and grain yield. This gene controls how much the rice plant can absorb nitrate from the soil. Interestingly, there are natural variations in this gene, with *indica* rice varieties having a version that leads to better growth and more tillers than *japonica* varieties. Overall, regulating *OsNPF5.16* expression holds promise for improving rice yields. The xylan glucuronosyltransferase gene (*OsGUX1*) is responsible for the addition of glucuronic acid residues to xylan and participates in the accumulation of cellulose and hemicellulose in the cell wall deposition, thereby thickening the primary cell walls of mesophyll cells, which might lead to reduced chlorophyll contents in rice leaves [97]. *OsGUX1* was downregulated under low light, which was higher in Swarnaprabha than in IR8, which corresponds to a greater accumulation of chlorophyll in Swarnaprabha than in IR8. *OsSCL1* is expressed in the elongating tissues of rice seedlings and the developing rice grain. Additionally, this is also thought to promote plant growth by regulating the expression of genes involved in cell elongation and starch synthesis [98]. We found a significant increment in plant height under LL conditions. The height of Swarnaprabha was relatively higher than that of IR8. This was reflected in parallel in the increased expression of *OsSCL1*, which was significantly more upregulated in Swarnaprabha than in IR8. *High tillering dwarf1 (HTD1)* encodes carotenoid cleavage dioxygenase, a homolog of *ArabidopsisMAX3*, which inhibits the growth of axillary buds and subsequent tiller formation in rice [99]. We found an overexpression of *HTD1* under LL conditions, which was higher in IR8 than in Swarnaprabha. This was correlated with the lowering of the tiller under LL parallelly. The expression patterns of the *OsROC1* gene are significantly upregulated under low-light stress up to the 10th day, and the expression value is higher in Swarnaprabha than in IR8. According to Ito et al. [100], *OsROC1* is an AGL2-type homeobox gene involved in protoderm differentiation and radial pattern formation during early rice embryogenesis. To date, there have been no specific reports detailing the impacts of these genes under low-light stress in rice, making them novel genes in rice under low-light stress conditions.

### 4.6. Pathway and Network Analysis of Selected Candidate Genes to Identify Hub Genes

The pathway and network analysis identified *OsAUX1*, *OsSBDCP1*, and *OsNPF5.16* as central hub genes in the regulatory network that governs rice’s response to low-light stress. These hub genes are crucial in coordinating hormonal signaling, carbohydrate metabolism, and nutrient assimilation pathways, which are essential for the plant’s adaptation to LL conditions. *OsAUX1* regulates auxin transport, which plays a central role in root and shoot growth by modulating cell elongation and division under LL stress [30]. The starch-binding domain-containing protein 1 (*OsSBDCP1*) is a key regulator of starch metabolism, maintaining energy production by controlling carbohydrate reserves during photosynthesis [101]. The nitrogen metabolism pathway in plants involves the uptake, transport, assimilation, and remobilization of nitrogen, which is essential for plant growth and development. The nitrate transporter *OsNPF5.16* ensures efficient nitrogen uptake and metabolism, enabling the plant to maintain nutrient balance and protein synthesis under LL conditions [96]. These genes form an interconnected network, making them prime targets for genetic improvement to enhance rice resilience to low-light environments.

## 5. Conclusions

Sixty-seven QTLs associated with grain yield and related traits under low-light and normal conditions were identified using recombinant inbred lines (RILs), developed from a cross between Swarnaprabha (low-light-tolerant) and IR8 (low-light-susceptible) varieties. Out of these QTLs, 33 were identified in both *Kharif* seasons. Twenty of these QTLs have not been previously reported under low-light conditions and are novel. These novel QTLs are associated with the days to 50% flowering, plant height, grain number, spikelet number, spikelet fertility percentage, panicle weight, thousand grain weight, and grain yield. We have also identified three QTL hotspots on chromosomes 1, 4, and 8. These hotspots contain QTLs for multiple traits. Twenty candidate genes were identified within these hotspots, and some of these genes were found to be differentially expressed between Swarnaprabha and IR8 under low-light stress conditions. These genes are potentially involved in auxin signaling, starch metabolism, sucrose biosynthesis, stress response, grain yield regulation, nitrate transport, chlorophyll content, cell wall thickening, plant height, tillering, and grain development. The identified three hub candidate genes, *OsAUX1*, *OsSBDCP1*, and *OsNPF5.16*, revealed vital hormonal and metabolic pathways aiding stress resilience. These findings provide new insights into the genetic mechanisms underlying low light tolerance in rice and could be used to develop rice varieties that are resilient to low-light stress conditions.

## Figures and Tables

**Table 1:** SSR and SNP markers used for a survey of polymorphism between parents the Swarnaprabha (tolerant) and IR8 (susceptible).

Chrom#	No. of SSR Markers Used	No. of SNP Markers Used	No. of Polymorphic SSR Markers	No. of Polymorphic SNP Markers Used	Total Number of Polymorphic Markers	Polymorphism %
1	153	50	11	9	20	9.85
2	151	20	6	4	10	6.43
3	170	21	12	5	17	7.85
4	111	24	2	4	6	4.44
5	105	20	4	5	9	6.45
6	98	22	7	5	12	10.83
7	76	15	5	5	10	9.89
8	92	12	14	3	17	16.35
9	67	14	5	5	10	12.35
10	40	9	0	2	2	2.04
11	61	9	5	1	6	11.43
12	59	8	4	0	4	7.46
**Total**	**1183**	**224**	**75**	**48**	**123**	**8.74**

**Table 2:** Stable QTLs associated with grain yield and related traits using the RIL mapping population developed from the cross between Swarnaprabha (tolerant) and IR8 (susceptible) in *Kharif* seasons of 2019 and 2021 under low-light (LL) and normal (control)-light (NL) conditions.

Sl. No.	Trait Name	QTL Name	Chromosome	Position (cM)	Flanking Markers	Physical Position (Mb)	LOD	PVE (%)	Additive	Condition	Season (*Kharif*)	Parental Contribution
1	DFF	*qDFF1.1_N_*	1	289–291	RM12276-RM10402	83.81–84.39	3.16–3.23	6.67–11.37	1.41–1.45	NL	2019, 2021	SP
2		*qDFF2.1_L_*	2	19–35	RM12601-TBGI090342	5.51–10.15	14.08–18.39	10.69–11.32	(−1.3–2.18)	LL	2019, 2021	IR8
3		*qDFF3.1_L_*	3	80–81	TBGI137429-RM14292	23.2–23.49	9.24–18.56	9.96–10.24	0.72–10.21	LL	2019, 2021	SP
4		*qDFF4.1_L_*	4	21–32	RM17478-TBGI210835	27.6–30.8	10.32–12.66	6.63–10.21	(−0.96–0.17)	LL	2019, 2021	IR8
5		*qDFF8.1_CLN_*	8	170–214	HYVSSR8-06-HYVSSR8-10	4.6–5.2	3.37–13.24	7.74–11.33	(−0.75-2.16)	NL, LL	2019, 2021	IR8
6	PH	*qPH1.1_CLN_*	1	27–32	TBGI054892-RM11935	7.83–37.7	3.34–12.57	5–10.82	0.35–0.55	NL, LL	2019, 2021	SP
7		*qPH1.2_CLN_*	1	100–104	RM11935-RM11940	37.7–37.8	4.06–5.85	4.76–9.58	8.50–10.51	NL, LL	2019, 2021	SP
8		*qPH1.3_CLN_*	1	158–164	RM11522-RM12182	45.82–47.56	5.01–13.18	4.16–11.22	2.60–3.94	NL, LL	2019, 2021	SP
9		*qPH3.1_N_*	3	6–7	RM15630-TBGI133686	1.74–2.03	7.88–8.43	8.86–11.25	0.87–1.13	NL	2019, 2021	SP
10		*qPH3.2_N_*	3	76–78	TBGI137429-RM14292	22.04–22.62	8.06–9.55	10.95–11.19	17.88–18.47	NL	2019, 2021	SP
11		*qPH3.3_N_*	3	320	RM14787-RM15981	92.8–92.9	14.13–14.64	6.95–9.21	2.14–3.05	NL	2019, 2021	SP
12		*qPH8.1_N_*	8	50–68	HYVSSR8-18-RM210	14.5–19.72	3.27–13.02	5.14–8.52	0.28–0.85	NL	2019, 2021	SP
13		*qPH8.2_N_*	8	12	RM556-TBGI341212	3.48–4.01	11.94–12.51	10.92–11.01	(−0.33–0.84)	NL	2019, 2021	IR8
14		*qPH9.1_CLN_*	9	83–102	TBGI390847-HYVSSR9-43	24.07–29.58	3.93–14.24	10.91–11.94	0.67–6.37	NL, LL	2019, 2021	SP
15	TN	*qTN1.1_N_*	1	79–85	TBGI054892-RM11935	22.91–37.7	3.69–3.98	12.47–13.75	(−0.39–1.39)	NL	2019, 2021	IR8
16	GN	*qGN1.1_L_*	1	100–107	RM11935-RM11940	37.7–37.8	3.01–4.89	11.83–12.69	0.51–9.78	LL	2019, 2021	SP
17	SN	*qSN1.1_L_*	1	101–103	RM11935-RM11940	37.7–37.8	3.68–4.01	11.20–15.21	2.23–8.73	LL	2019, 2021	SP
18		*qSN3.1_CLN_*	3	278–292	RM14787-RM15981	80.62–84.68	3.05–3.95	11.28–17.70	0.08–10.34	NL, LL	2019, 2021	SP
19	SFP	*qSFP1.1_N_*	1	80–85	TBGI054892-RM11935	37.2–37	3.46–4.39	10.08–11.65	(−0.03–1.20)	NL	2019, 2021	IR8
20		*qSFP1.2_N_*	1	157–159	RM11522-RM12182	45.53–46.11	3.72–4.39	6.05–10.09	0.25–1.005	NL	2019, 2021	SP
21		*qSFP1.3_N_*	1	53–54	RM10207-TBGI031980	15.37–15.66	4.33–7.43	7.19–8.25	(−0.4–0.16)	NL	2019, 2021	IR8
22		*qSFP3.1_N_*	3	111–120	TBGI137429-RM14292	32.19–34.8	2.73–2.80	5.21–9.29	1.29–2.84	NL	2019, 2021	SP
23		*qSFP4.1_L_*	4	13–72	RM17487-RM17478	31.1–30.8	3.18–5.28	7.14–8.34	0.26–1.27	LL	2019, 2021	SP
24		*qSFP6.1_N_*	6	32–53	RM20659-RM20372	9.28–15.37	3.13–3.23	5.92–7.32	(−0.45–1.03)	NL	2019, 2021	IR8
25		*qSFP6.2_N_*	6	205–207	TBGI278662-TBGI273346	59.45–60.03	2.99–3.94	10.11–10.24	0.54–0.55	NL	2019, 2021	SP
26	PW	*qPW1.1_CLN_*	1	85–108	RM11935-RM11940	37.7–37.8	3.10–3.99	6.50–14.52	0.19–0.24	NL, LL	2019, 2021	SP
27		*qPW3.1_L_*	3	188–215	RM545-RM14772	54.52–62.35	3.48–3.58	11.74–12.11	0.04–0.15	LL	2019, 2021	SP
28	TGW	*qTGW1.1_L_*	1	102–103	RM11935-RM11940	37.7–37.8	3.50–4.17	6.44–7.55	0.45–0.96	LL	2019, 2021	SP
29		*qTGW1.2_L_*	1	158–207	RM11522-RM12182	45.82–60.03	3.40–6.12	10.17–10.21	0.09–0.13	LL	2019, 2021	SP
30		*qTGW4.1_L_*	4	12–49	RM17478-TBGI210835	27.6–30.8	2.76–4.89	10.59–11.22	(−0.30–0.06)	LL	2019, 2021	IR8
31		*qTGW12.1_L_*	12	8–10	RM27824-RM28524	2.32–2.9	3.06–5.65	7.91–8.56	0.56–0.58	LL	2019, 2021	SP
32	GY	*qGY7.1_CLN_*	7	81–125	TBGI322578-RM21808	23.49–36.25	2.89–3.35	15.08–17.06	1.16–1.84	NL, LL	2019, 2021	SP
33		*qGY8.1_CLN_*	8	161–201	HYVSSR8-06-HYVSSR8-10	4.6–5.2	2.52–4.18	11.27–18.78	0.15–0.24	NL, LL	2019, 2021	SP

DFF: days to 50% flowering; PH: plant height (cm); TN: tiller number; GN: grain number per panicle; SN: spikelet number per panicle; SFP: spikelet fertility percentage; PW: panicle weight (g); TGW: thousand grain weight (g); GY: grain yield per plant (g).

**Table 3:** QTL hotspots identified for grain yield and related traits using RIL mapping population developed from the cross between Swarnaprabha (tolerant) and IR8 (susceptible) in *Kharif* seasons of 2019 and 2021 under low-light (LL)and normal (control)-light (NL) conditions.

Scheme	QTL Cluster No.	Chrom#	Marker Interval	Position (Mb) for Flanking Markers	Peak Interval (cM)	Window Size (Mb)	No. of QTLs	Name of the QTLs	Traits	No. of Genes	No. of Candidate Genes
1	I	1	RM11935-RM11940	37.7–37.8	85–108	0.16	5	*qPH1.2_CLN_*, *qGN1.1_L_*, *qSN1.1_L_*, *qPW1.1_CLN_*, *qTGW1.1_L_*	PH, GN, SN, PW, TGW	239	17
2	II	4	RM17478-TBGI210835	27.6–30.8	12–49	3.2	3	*qDFF4.1_L_*, *qSFP4.1_L_*, *qTGW4.1_L_*	DFF, SFP, TGW	516	1
3	III	8	HYVSSR8-06-HYVSSR8-10	4.6–5.2	161–204	0.6	2	*qDFF8.1_CLN_*, *qGY8.1_CLN_*	DFF, GY	171	2

DFF—days to 50 % flowering; PH—plant height (cm); GN—grain number per panicle; SN—spikelet number per panicle; SFP—spikelet fertility percentage; PW—panicle weight (g); TGW—1000 grain weight (g); GY—grain yield per plant (g).

## Data Availability

The original contributions presented in this study are included in the article Further inquiries can be directed to the corresponding author.

## Supplementary Materials

The following supporting information can be downloaded at: https://www.mdpi.com/article/10.3390/biom15101388/s1, Figure S1: Identification of candidate hub genes (*OsAUX1*, *OsSBDCP1*, and *OsNPF5.16*) using the Hub Gene Network based on functional annotation of the co-expressed genes; Figure S2: Starch and sucrose metabolism pathway in relation to starch biding domain-containing protein 1 (*OsSBDCP1*) candidate hub gene with amylase trait depicted as red line; Figure S3: Plant hormone signal transduction pathway related to candidate hub gene *OsAUX1*; Figure S4: Nitrogen metabolism pathway related with Nitrate Transporter 1/Peptide Transporter 5.16 (*OsNPF5.16*) candidate hub gene; Table S1: SSR Markers; Table S2: Polymorphic SNP markers; Table S3: Descriptive statistics; Table S4: ANOVA; Table S5: Linkage map; Table S6: All the identified QTLs-R; Table S7: Candidate genes; Table S8: RT-PCR Primers;
