# Supplementary material for: Identification of Novel Quantitative Trait Loci and Candidate Genes Associated with Grain Yield and Related Traits Under Low-Light Stress Conditions in Rice
Source: Biomolecules. 2025 Sep 29;15(10):1388. doi: 10.3390/biom15101388 (PMC12562436; doi:10.3390/biom15101388)
Supplement: Supplementary file 1 [file biomolecules-15-01388-s001.zip › biomolecules-3608205-revise-Supplementary.pdf]

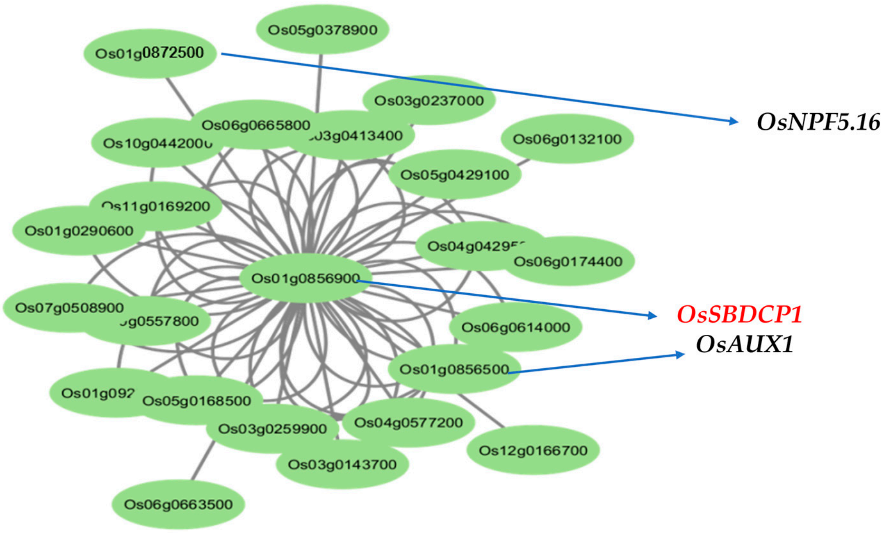

Supplementary Figure S1. Identification of candidate hub genes *(OsAUX1, OsSBDCP1,* and *OsNPF5.16)* using the Hub Gene Network based on functional annotation of the co-expressed genes.

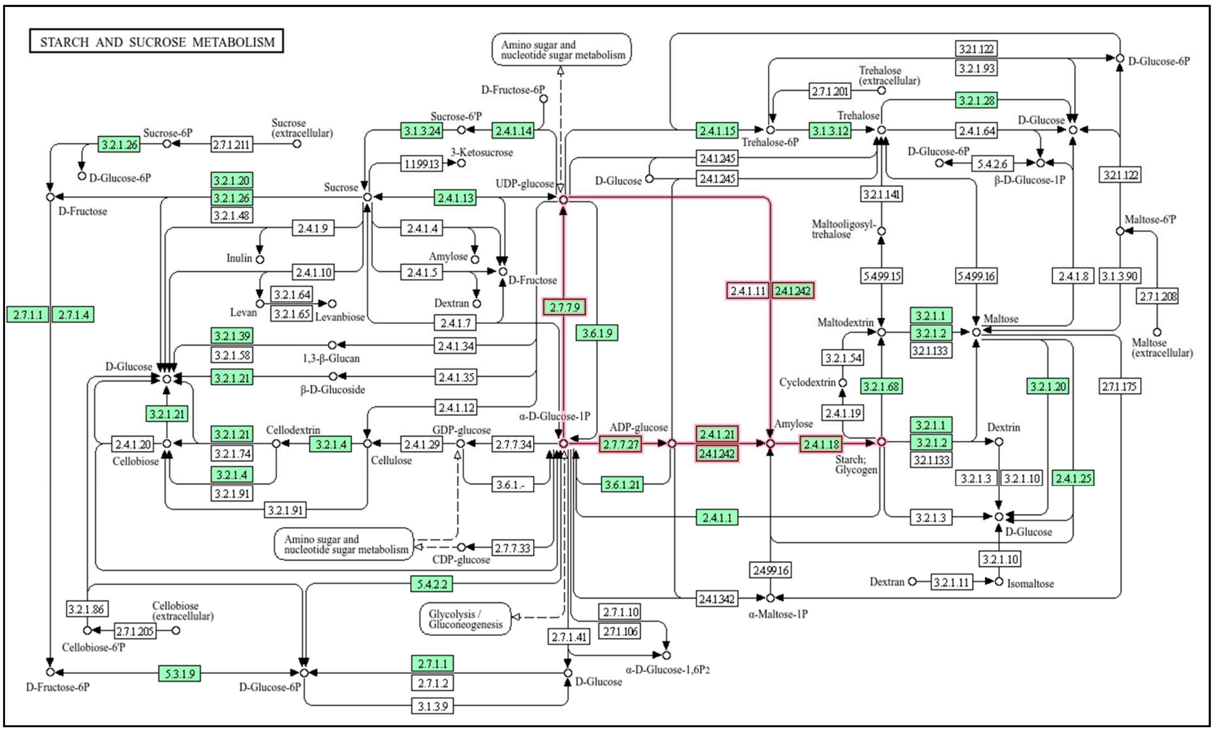

Supplementary Figure S2. Starch and sucrose metabolism pathway in relation to starch biding domain-containing protein 1 (*OsSBDCP1)* candidate hub gene with amylase trait depicted as red line.

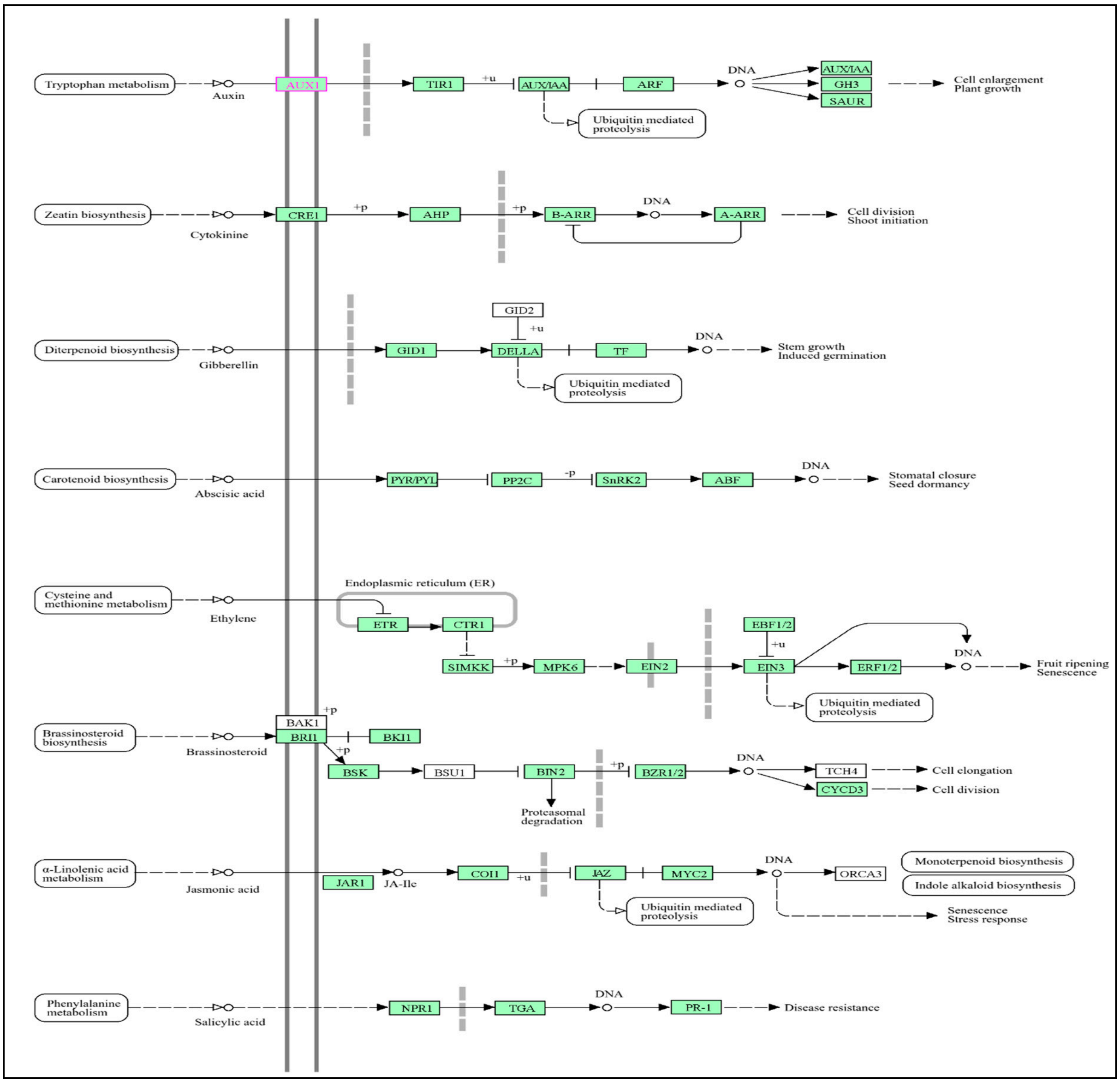

Supplementary Figure S3. Plant hormone signal transduction pathway related to candidate hub gene *OsAUX1* (in red).

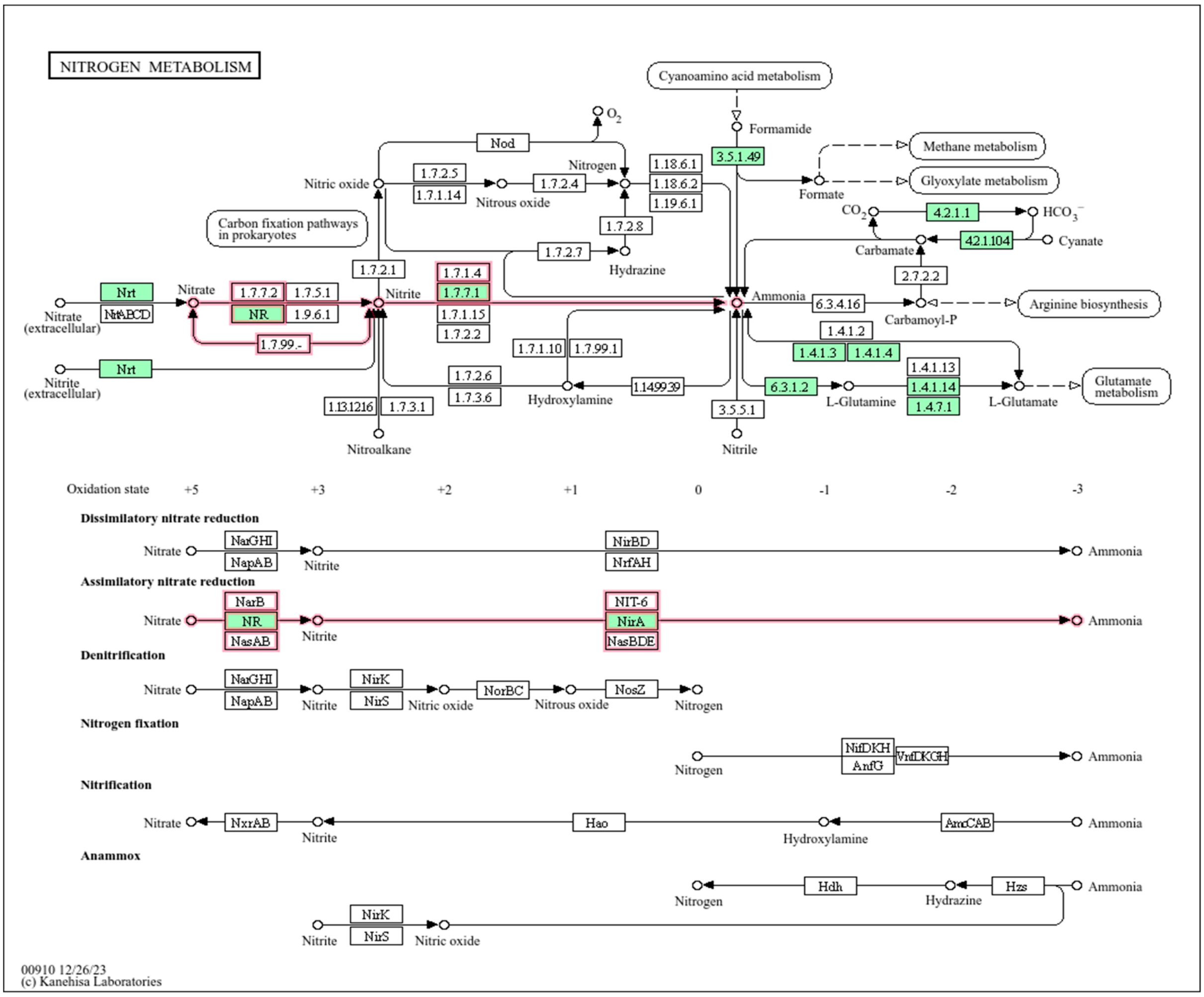

Supplementary Figure S4. Nitrogen metabolism pathway related with Nitrate Transporter 1/ Peptide Transporter 5.16 (*OsNPF5.16*) candidate hub gene.
